# Querschnittstudie zum Versorgungsstand von Familien nach Suizid und Suizidversuch eines Elternteils in Bayern

**DOI:** 10.1007/s11553-022-00981-x

**Published:** 2022-09-19

**Authors:** Simon Finkeldei, Tita Kern, Susanna Rinne-Wolf

**Affiliations:** 1 AETAS Kinderstiftung, München, Deutschland; 2 Fakultät für Sport- und Gesundheitswissenschaften, Technische Universität München, Uptown München-Campus D, Georg-Brauchle-Ring 60/62, 80992 München, Deutschland

**Keywords:** Versorgung, Kinder, Krise, Beratung, Trauma, Health services, Children, Crisis, Counselling, Trauma

## Abstract

**Hintergrund:**

In Bayern wurde 2019 mit 1520 Fällen die höchste absolute Anzahl von Suiziden in Deutschland registriert. Suizid als Todesursache ist besonders belastend für Angehörige und das Risiko von betroffenen Kindern, selbst im weiteren Lebensverlauf Suizid zu begehen, ist signifikant erhöht. Frühe und spezifische Ansätze der sog. psychosozialen Notfallversorgung sind nach hoch belastenden Lebenserfahrungen, wie Suizid im Nahfeld, fachlich indiziert.

**Ziel der Arbeit:**

Ziel der Querschnittstudie ist es, die Versorgungssituation von Familien nach Suizid und Suizidversuch eines Elternteils in Bayern zu erheben und dadurch Bedarfe zu erkennen.

**Methoden:**

Daten zu Versorgungsangeboten, der (Selbst)einschätzung der Kompetenzen zu traumaspezifischen Aspekten und der grundsätzlichen Beurteilung der Versorgungssituation und -qualität in Bayern wurden bei 108 Jugendämtern und Beratungsstellen per telefonischer Befragung erhoben und deskriptiv ausgewertet.

**Ergebnisse:**

Fälle von Suizid/-versuch kommen in der Beratungs- und Betreuungsrealität von Mitarbeitenden in Jugendämtern und Beratungsstellen vor. Die am häufigsten genannte Hilfe ist die Weitervermittlung in andere zumeist heilkundliche Angebote. 80 % der befragten Mitarbeitenden in Beratungsstellen und Jugendämtern halten die Einführung einer zentralen Notfallrufnummer für Familien und Fachkräfte für sinnvoll.

**Schlussfolgerung:**

Fachkräfte sehen den Bedarf für Beratung bei den Betroffenen und wollen diesem auch in der eigenen Einrichtung entsprechen, fühlen sich jedoch in Folge begrenzter interner und externer Angebote sowie eigener Qualifikation dafür nicht ausreichend ermächtigt. Die Angebote, in die weitervermittelt wird, sind in den meisten Fällen weder spezifisch, passgenau noch kurzfristig verfügbar, was angesichts des hohen Erkrankungsrisikos der betroffenen Kinder und Jugendlichen einen kritischen Faktor darstellt. Es besteht ein Bedarf für unmittelbar erreichbare spezifische Unterstützung sowohl für Fachkräfte als auch für betroffene Familien, z. B. durch eine Notfallrufnummer.

## Abstract (en)

**Background:**

In 2019 the highest absolute number of suicides in Germany was registered in Bavaria, with 1520 cases. Suicide as a cause of death is particularly stressful for relatives and the risk of affected children committing suicide themselves later in life is significantly increased. Early and specific approaches of so-called psychosocial emergency care are professionally indicated after highly stressful life experiences, such as the suicide of a close person.

**Objective:**

The aim of the cross-sectional study is to assess the services available for families after suicide and attempted suicide of a parent in Bavaria and thus to identify the needs.

**Methods:**

Data on services, the (self)assessment of competences on trauma-specific aspects and the general assessment of the care situation and quality in Bavaria were collected from 108 youth welfare offices and counselling centers by telephone survey and analyzed descriptively.

**Results:**

Cases of suicide and attempted suicide occur in the counselling and care reality of staff in youth welfare offices and counselling centers. The most frequently mentioned help is referral to other mostly curative services. Of the staff interviewed in counselling centers and youth welfare offices, 80% consider the introduction of a central emergency telephone number for families and professionals to be sensible.

**Conclusion:**

Professionals see the need for counselling among those affected and want to meet this need in their own institution but do not feel sufficiently empowered to do so due to limited internal and external offers as well as their own qualifications. In most cases, the services to which referrals are made are not specific, suitable, or available at short notice, which is a critical factor in view of the high risk of illness among the children and adolescents concerned. There is a need for immediately accessible specific support for both professionals and affected families, e.g. through an emergency telephone number.

Laut WHO liegt in Ländern mit einem hohen durchschnittlichen Haushaltseinkommen wie in Deutschland die Prävalenz von Suizidversuchen bei 3 pro 1000 erwachsenen Einwohner*innen [30]. Im Jahr 2019 verstarben in Deutschland insgesamt 9041 Personen durch Suizid [26]. Das waren über 25 Personen pro Tag. In Bayern wurde 2019 mit 1520 Fällen die höchste absolute Anzahl von Suiziden in Deutschland registriert, wodurch Bayern sich bezogen auf die Gesamteinwohnerzahl an 10. Stelle im Bundesvergleich befindet [10]. Die Zahl der nicht primär tödlich endenden Suizidversuche sowie geschätzte Dunkelziffern steigern den Umfang dieser tragischen Thematik um ein Vielfaches. Die Deutsche Depressionshilfe schätzt, dass die Suizidversuche die Suizide um den Faktor 15–20 übersteigen [7]. Männer nehmen sich deutlich häufiger das Leben als Frauen, rund 76 % der Selbsttötungen wurden von Männern begangen. Die meisten Männer, die sich suizidieren, sind im Alter zwischen 50 und 55 Jahren alt [26]. Das ist eine Altersgruppe, in der viele dieser Männer eine Familie haben und gemeinsam mit ihren Kindern bzw. Jugendlichen in einem Haushalt leben. Nicht zuletzt dadurch erhält diese selbstbezogene Form von Gewalt auch eine interpersonelle Dimension. Häufig zählen Kinder und Jugendliche zu den Angehörigen und Hinterbliebenen, teilweise zu Augenzeug*innen und manchmal zu direkt Betroffenen. Auch unabhängig von der Frage, ob Kinder oder Ehepartner*innen die suizidierte Person gefunden haben, stellen Suizide eine in besonderem Maß hoch belastende Lebenserfahrung dar [22, 25]. Studien zeigen, dass Kinder von Menschen, die durch Suizid versterben, ein erhöhtes Risiko für psychische Erkrankungen aufweisen. Dazu gehören Depressionen, Psychosen und Persönlichkeitsstörungen [28]. Zusätzlich ist das Risiko der Kinder und Jugendlichen, selbst im weiteren Lebensverlauf Suizid zu begehen, signifikant erhöht [11, 14]. Beispielsweise ist im Vergleich zu Kindern, die ein Elternteil bei einem Unfall verloren haben, das Risiko von suizidbetroffenen Kindern um 82 % erhöht [14]. Besonders gefährdet sind Kinder, die beim Suizid des Elternteils unter 6 Jahre alt waren [28]. Wie ein solches hoch belastendes, gewaltassoziiertes Indexereignis individuell verarbeitet wird, ist allerdings von verschiedenen Faktoren abhängig: Zunächst entscheiden sog. Schutz- und Risikofaktoren darüber, wie Traumareaktionen ausfallen [8]. Darüber hinaus ist die Verarbeitung davon abhängig, welche Ereignisse und Erfahrungen in der Folgezeit nach dem Suizid(versuch) gemacht werden [8]. Das Fehlen sozialer Unterstützung nach einem traumatischen Lebensereignis wurde bereits 2003 als wesentlicher Faktor, der sich prognostisch ungünstig auf die Verarbeitung bei Betroffenen auswirkt, herausgearbeitet [3]. Der gleiche Effekt ist für sog. „post trauma life stress“ nachgewiesen [3] – also für auf das eigentliche Ereignis folgende weitere Belastungsfaktoren. Darüber hinaus sind das Verhalten von nahestehenden Bezugspersonen gegenüber betroffenen Kindern und Jugendlichen und die Art der Bewältigungsbemühungen wesentlich [13, 27]. Dem Einfluss Rechnung tragend, den diese Faktoren auf die Art und Möglichkeiten der frühen Verarbeitung direkt nach dem Indexereignis insbesondere für Kinder und Jugendliche haben [13, 16, 27], geben die Handlungsleitlinien der Fachgesellschaften wie bspw. die S2K-Leitlinien zu akuten Folgen psychischer Traumatisierung oder die Empfehlungen des Bundesamtes für Bevölkerungsschutz und Katastrophenhilfe eine frühe und spezifische Akuthilfe bereits unmittelbar nach hoch belastenden Lebenserfahrungen vor [2, 4]. Aufgrund der Häufigkeit von Suizid und Suizidversuch nehmen diese im Rahmen der hoch belastenden Lebenserfahrungen eine prominente Stelle ein.

Ziel dieser Querschnittstudie ist es, die Versorgungssituation/den Versorgungsstand bezüglich der indizierten frühen und spezifischen Akuthilfe von Suizid/-versuch betroffener Familien zu erheben und dadurch offen bleibende Bedarfe zu erkennen. Als wesentliche Teile der Versorgung von Familien wurde in diesem ersten Schritt der Fokus auf Angebote von Erziehungsberatungsstellen und Jugendämtern gelegt. Dies ist insbesondere darin begründet, dass laut § 69 Abs. 3 SGB VIII allerorts Jugendämter eingerichtet sein müssen und dass sowohl die Jugendämter als auch die Beratungsstellen einen gesetzlich festgeschriebenen Versorgungsauftrag für Kinder, Jugendliche und Familien (§ 17 SGB VIII; § 28 SGB VIII) inne haben. Die hier beschriebene Studie ist Teil des durch das Bayerische Staatsministerium für Familie, Arbeit und Soziales geförderten Forschungs- und Versorgungsprojekt „Kurswechsel“, welches die Folgen zwischenmenschlicher und selbstbezogener Gewalt [29] untersucht.

## Methode

### Stichprobe und Vorgehen

Vier bis sechs Wochen vor der Befragung wurden die Sprecher*innen der Jugendämter der Regierungsbezirke sowie der Bundeskonferenz für Erziehungsberatung e. V. über die bevorstehende Studie informiert und ihnen per E‑Mail Informationen zur Verfügung gestellt, mit der Bitte, als Multiplikator*innen tätig zu werden. Für die Erhebung wurden alle Jugendämter sowie eine Stichprobe der Beratungsstellen in Bayern kontaktiert. Für die Jugendämter fand eine Vollerhebung in den 7 bayerischen Regierungsbezirken statt. Für die Beratungsstellen wurde eine Auswahl getroffen. Berücksichtigt wurden Beratungsstellen in allen kreisfreien Städten. Darüber hinaus wurde anhand einer zweistufigen Clusterstichprobe eine Stichprobe aus den Regierungsbezirken bzw. Landkreisen generiert. Als Einrichtungsarten wurden Erziehungsberatungsstellen, Ehe‑, Familien- und Lebensberatungsstellen, spezialisierte Facheinrichtungen wie bspw. Beratungsstellen mit Schwerpunkt Suizidprävention sowie sonstige Beratungsstellen einbezogen.

Bei dieser Erhebung handelt es sich um eine Querschnittstudie. Sie fand mittels einer telefonischen Befragung statt. Dafür wurde ein selbst entwickelter teilstandardisierter Fragebogen mit 30 Fragen verwendet. Dieser wurde als Eingabemaske für die Interviewer*innen auf der Plattform SoSci Survey (Version 3.2.43) programmiert. Es gab keine finanziellen oder sonstigen Anreize zur Teilnahme an der Befragung. Der Fragebogen wurde im Vorfeld im Hinblick auf Sprache, Verständlichkeit und Logik getestet. Anhand der Rückmeldungen wurde die Umfrage dann entsprechend überarbeitet. Im Pre-Test betrug die Zeit für das Ausfüllen des Fragebogens durchschnittlich 20 min. Zur besseren Verständlichkeit werden Ergebnisse hier in anderer Reihenfolge präsentiert, als sie in der Umfrage erhoben wurden.

Die Interviewer*innen wurden in der Verwendung von SoSci Survey sowie für die Befragungen in mehreren Sitzungen geschult, um die größtmögliche Einheitlichkeit in der Befragung zu gewährleisten. Vor Beginn der Befragung wurde dem Teilnehmenden das Thema des verwendeten Fallbeispiels (elterlicher Suizid) offengelegt und ihr Einverständnis („informed consent“) eingeholt. Ebenso wurde auf den Datenschutz hingewiesen und darauf, dass keine Aufzeichnung der Gespräche, aber eine digitale Eingabe in SoSci Survey erfolgen.

Erhobene Daten der Teilnehmenden wurden anonymisiert und so verarbeitet, dass keine Rückschlüsse auf die jeweiligen Personen gezogen werden können. Alle Teilnehmenden wurden hierüber und über die Freiwilligkeit sowie die erforderliche Zeit für die Beantwortung aller Fragen informiert. Eine gesonderte ethische Begutachtung wurde nicht durchgeführt, da die Umfrage in Übereinstimmung mit der Deklaration von Helsinki stattfand.

### Fragebogen

Der Fragebogen bestand aus Single- und Multiple-choice- sowie offenen Fragen. Es bestand sowohl die Möglichkeit, Fragen unbeantwortet zu lassen, als auch bei diversen Fragen die Möglichkeit von Mehrfachnennungen. Dadurch ergeben sich pro Frage sowohl die Möglichkeit einer verschiedenen Anzahl an beantwortenden Personen als auch eine verschiedene Anzahl von Antworten. Inhaltliche Berücksichtigung fanden die Schwerpunkte:

- Versorgungssituation von betroffenen Familien,
- (Selbst)einschätzung der Kompetenzen zu traumaspezifischen Aspekten und
- die grundsätzliche Beurteilung der Versorgungssituation und -qualität in Bayern.

Die Fragen zur Versorgungssituation und deren Beurteilung durch die Befragten wurden anhand eines konkreten Fallbeispiels konstruiert, das sich auf den Suizidversuch eines Familienvaters bezieht (vgl. Infobox). Das Fallbeispiel entspricht einem anonymisierten Realfall.

#### Infobox „Fallbeispiel Befragung Jugendämter und Beratungsstellen“

Ein Vater, 44 Jahre alt, versucht sich zu suizidieren und stranguliert sich im Kinderzimmer seines Sohnes Tim. Tim, 8 Jahre alt, hört den Krach eines umfallenden Stuhls und geht darauf in sein Kinderzimmer. Dort findet er den Vater und verständigt sofort die Mutter, 40 Jahre alt. Diese alarmiert den Rettungsdienst. Der Vater überlebt und wird zur weiteren medizinischen Versorgung in das nächste Klinikum gebracht. Zur Familie gehört noch die Tochter Mia, 3 Jahre alt. Die Familie lebt in Ihrem Einzugsgebiet.

Neben der Erfassung der Inhalte des Fragebogens wurden durch die eingesetzten Interviewer*innen auch die Erreichbarkeit der Einrichtungen sowie die Anzahl der hausinternen Weitervermittlungen dokumentiert. Durch diese Dokumentation kann dargestellt werden, wie viele Kontaktversuche notwendig waren, um eine Einrichtung zu erreichen.

### Datenanalyse

Nach Abschluss der Datenerhebung wurden die erhobenen Daten mithilfe der statistischen Analysesoftware SPSS® für Windows (Version 25.0.0.1, IBM, 2017, Armonk, NY, USA), MAXQDA Standard 2020 (Version 20.4.0, VERBI GmbH, Berlin) und Excel analysiert. Als deskriptive Statistiken wurden absolute und relative Häufigkeiten bei kategorialen Variablen angeben.

## Ergebnisse

Die Anzahl (*n*) der Stichproben betrug 194. Alle Einrichtungen in der gewählten Stichprobe wurden im Zeitraum vom 14.09.2020 bis zum 20.11.2020 zu üblichen Telefonzeiten und an verschiedenen Werktagen kontaktiert. 32,4 % der Einrichtungen konnten erst beim 2. bis 6. Anrufversuch erreicht werden. Insgesamt waren 10,4 % der Beratungsstellen und 3,1 % der Jugendämter trotz 6‑maliger Anrufversuche gar nicht (auch nicht per Anrufbeantworter o. Ä.) erreichbar. Es konnten 181 (93,3 %) der 194 Einrichtungen telefonisch erreicht und 108 (56 %) Personen befragt werden. Von diesen Personen beantworteten alle den Fragebogen bis zum Ende, jedoch nicht alle Personen auf alle Fragen, wodurch teilweise unterschiedliche *n* bei den Antworten entstehen.

Der überwiegende Teil der Teilnehmenden war im Jugendamt (41,9 %) oder in Beratungsstellen (53,3 %) tätig. Mehr als zwei Drittel der befragten Personen bekleidete eine Leitungsposition in der Einrichtung (70,47 %). Eine genaue Übersicht über die Arten der befragten Einrichtungen sowie die Art der Leitungsposition der befragten Personen findet sich in Tab. 1. Die befragten Personen verfügten über durchschnittlich 13,396 Jahre Berufserfahrung (*SD* = 10,955) mit einem Median von 9 Jahren.

**Tab. 1:** Befragte Einrichtungen und Personen

Befragte Einrichtungen und Personen	
*n*	108
*Einrichtungsart*	
Jugendamt/kommunale Einrichtung	44 (40,7 %)
Beratungsstelle	56 (51,9 %)
Krisendienst	1 (0,93 %)
Sonstiges	4 (3,70 %)
Keine Antwort	3 (2,78 %)
*Position*	
(Stellen)leitung, Leitung ASD	53 (49,07 %)
Stlv. Leitung	4 (3,70 %)
Abteilungs‑/Gruppenleitung	17 (15,74 %)
Sonstige	31 (28,70 %)
Keine Antwort	3 (2,78 %)

### Versorgungssituation von betroffenen Familien

Es haben 84,11 % der Antwortenden im Rahmen ihrer Tätigkeit mit Menschen, wie im Fallbeispiel, zu tun. Auf die Frage, wie schnell, wie oft und wie lang der Familie Hilfe in der eigenen Einrichtung angeboten werden kann, zeigte sich anhand der insgesamt 82 erhaltenen Antworten, dass eine zeitnahe Versorgung ohne zeitliche Limitationen möglich ist. 19,5 % gaben an, Hilfe sofort bzw. noch am selben Tag anbieten zu können. 25,6 % können innerhalb von 2 bis 4 Tagen Hilfe anbieten und 30,5 % innerhalb einer Woche. Das bedeutet, dass nur etwa ein Viertel der antwortenden Personen angab, dass Hilfe innerhalb von 2 oder mehr Wochen möglich sei. Darüber hinaus zeigen die Daten, dass der Großteil der Antwortenden, dem gesetzlichen Auftrag folgend, eine Versorgung der Familie ermöglichen kann. 84,2 % gaben an, dass es keine Limitationen hinsichtlich der Häufigkeit für Betreuungstermine und 89,3 % gaben an, dass es keine Limitationen hinsichtlich des Zeitraums für eine Betreuung gibt, sondern diese sich an den Bedarfen und Bedürfnissen der Familie orientiert.

**Abb. 1:**
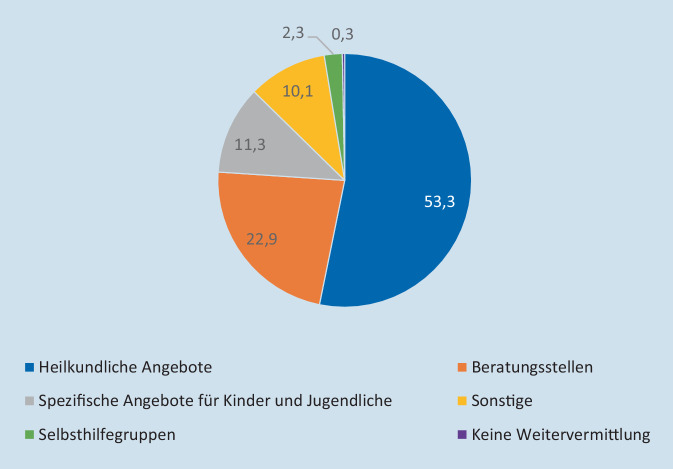
An welche Stellen können Sie die Familien in Ihrer Region verweisen?

Bezüglich der Versorgungsituation der betroffenen Familie wurden sowohl die Möglichkeiten in den befragten Einrichtungen selbst als auch Verweismöglichkeiten abgefragt. Die genannten grundsätzlichen Verweismöglichkeiten ergaben ein deutlich zur heilkundlichen Versorgung tendierendes Bild (Abb. 1).

Während über 95 % der Einrichtungen angaben, der Mutter im Fallbeispiel Hilfe in der eigenen Einrichtung anbieten können, nimmt diese Hilfe zu knapp 50 % die Form einer Weitervermittlung an andere Stellen an. Für das ältere Kind (8 Jahre) sind es etwas über 90 % der Einrichtungen, die Hilfe anbieten können, allerdings besteht diese auch zu knapp 40 % aus Weitervermittlung. In Bezug auf das jüngere Kind (3 Jahre) bieten knapp 80 % Hilfen an, diese zu 28 % in Form von einer Weitervermittlung. Für alle 3 betroffenen Personen war die Weitervermittlung das am häufigsten genannte Hilfsangebot.

Ein differenzierterer Blick auf die Weitervermittlung zeigt, dass diese am häufigsten in heilkundliche Angebote wie Psychiater*innen, Psychotherapeut*innen und klinische Einrichtungen erfolgt. Während dies in bis zu 32,6 % bei der Mutter zutraf, so waren es beim älteren Kind schon 46,5 % und beim jüngeren Kind 37,5 %. Traumaspezifische Angebote wurden in Bezug auf alle 3 in weniger als 10 % der Fälle genannt. Nur 25 von 105 antwortenden Personen (23,8 %) gaben an, spezifische Facheinrichtungen oder Beratungsstellen für Kinder nach Suizid oder Suizidversuch im Nahfeld in der eigenen Region zu haben. Diese Angebote bestanden zu 39 % in heilkundlichen Angeboten inklusive klinischer Einrichtungen, spezifische Beratungsstellen wurden insgesamt nur 5‑mal genannt.

Weitere Arten der Hilfe für die Familie im Fallbeispiel wurden, differenziert nach Betroffenen, wie folgt benannt: Für die Mutter konnten primäre Allgemeinversorgung (Bedarfsermittlung/Clearing, Erstberatung/Überbrückungsberatung und Psychoedukation: 19,4 %), Versorgung mit Fokus auf die Mutter selbst (allgemeine Beratung, Trauerberatung, Ehe‑/Paarberatung, traumatherapeutische Beratung und Resilienz- und Ressourcenarbeit: 32,1 %) sowie Hilfen mit Fokus auf die Kinder (Erziehungsberatung und Eltern-Kind-Beratung: 11,2 %) angeboten werden. Hierbei wurde bei 294 einzelnen Antworten die traumatherapeutische Beratung insgesamt nur 5‑mal benannt. Für den 8‑Jährigen wurden primäre Allgemeinversorgung (Bedarfsermittlung/Clearing, Erstberatung/Überbrückungsberatung, Psychoedukation und Diagnostik: 12,7 %), Versorgung mit Fokus auf den 8‑Jährigen selbst (Einzelberatung, Erziehungsberatung über die Mutter, Familiengespräche, Einbindung des Umfelds wie Schule und Angehörige, traumatherapeutische Beratung und Ressourcenarbeit/Ressourcenstärkung: 21,9 %), allgemeine Angebote für Kinder (Spieltherapie, Erlebnispädagogik, Gruppenangebote und unspezifische Angebote für Kinder: 8,8 %) sowie Angebote der Kinder- und Jugendhilfe nach SGB VIII (z. B. Erziehungsbeistandschaft, Hilfen zur Erziehung, Eingliederungshilfe etc.: 16,3 %) genannt. Bei insgesamt 251 einzelnen Antworten wurde die traumatherapeutische Beratung nur 4‑mal genannt. Die Hilfen für die 3‑Jährige umfassten primäre Allgemeinversorgung (Bedarfsermittlung/Clearing und Diagnostik: 4,2 %), Versorgung mit Fokus auf die 3‑Jährige selbst (Spieltherapie und Einbindung des Umfelds: 14,7 %), Versorgung über einen Fokus auf die Mutter (ausschließliche Versorgung der Mutter, Beratung/Betreuung der Mutter, Stärkung der Mutter und finanzielle Entlastung der Mutter: 21,0 %), gemeinsame Versorgung der 3‑Jährigen und der Mutter („case management“, Mutter-Kind-Gruppe, Familiengespräch/Familientherapie, Erziehungsberatung für Kleinkinder und Eltern-Kind-Beratung: 12,6 %) sowie Angebote der Kinder- und Jugendhilfe nach SGB VIII (z. B. sozialpädagogische Familienhilfe, Tagesbetreuung etc.: 14,7 %) genannt. Die traumatherapeutische Beratung wurde nicht genannt.

**Abb. 2:**
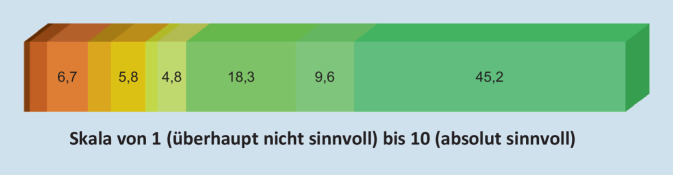
Für wie sinnvoll halten Sie eine zentrale Notfallnummer für Familien und Fachkräfte?

Nach Verbesserungsvorschlägen zur Versorgungssituation von Familien wie im Fallbeispiel befragt, gaben 87,2 % der Antwortenden an, Bedarfe zu erkennen. Mehr als ein Drittel der Nennungen (35,1 %) bezogen sich dabei auf den quantitativen Ausbau der Versorgung, während 21,6 % die Verbesserung der Öffentlichkeitsarbeit (inklusive Bekanntmachung von Suizidprävention und Psychoedukation) und 11,5 % die verbesserte Aus‑, Fort- und Weiterbildung von Fachkräften betrafen. Die Einführung einer zentralen Notfallrufnummer für Familien und Fachkräfte hielten knapp 80 % der Befragten für eher bis absolut sinnvoll, nur eine Person befand dies für überhaupt nicht sinnvoll (Abb. 2).

### (Selbst)einschätzung der Kompetenzen zu traumaspezifischen Aspekten

Von den befragten Fachkräften gaben 58,1 % an, dass das Thema „akuter traumatischer Stress bei Kindern und Jugendlichen“ während ihrer Aus‑, Fort- und Weiterbildung nicht behandelt wurde.

**Abb. 3:**
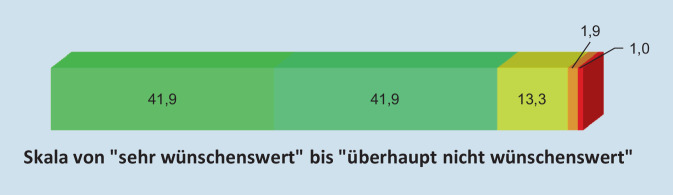
Wie sehr sind weitere Kompetenzen im Bereich der akuten Unterstützung für Kinder und Jugendliche nach potenziell traumatisierenden Erlebnissen wünschenswert?

Die Fragen nach Aus‑, Fort- und Weiterbildung waren offen gestellt, so dass pro Person mehrere Antworten möglich waren, die im Anschluss kategorisiert wurden. Bei den Fachkräften, die das Thema behandelt hatten, geschah dies entweder im Rahmen ihres Studiums (10,8 %), während einer Therapieausbildung (22,9 %), während einer fachspezifischen Aus‑, Fort- oder Weiterbildung (35,1 %) oder in anderen Seminaren und Fortbildungen (31,1 %). Ein psychotraumatologischer Fokus der fachspezifischen Aus‑, Fort- oder Weiterbildung auf Kinder und Jugendliche wurde von nur 4 Personen genannt. 83,8 % der Befragten hielten die Weiterentwicklung der eigenen Kompetenzen im Bereich der akuten Unterstützung für Kinder und Jugendliche nach potenziell traumatisierenden Erlebnissen für wünschenswert. Nur 2,9 % hielten dies für eher oder überhaupt nicht wünschenswert (Abb. 3).

### Grundsätzliche Beurteilung der Versorgungssituation und -qualität in Bayern

Den Befragten wurde neben spezifischen Fragen zur Versorgung der im Fallbeispiel dargestellten Familie nach Suizidversuch auch allgemeine Fragen zur Versorgungs- und Angebotslandschaft nach belastenden Notfällen gestellt. So wurden sie zunächst gefragt, ob in ihrer Region weiterführende Hilfsangebote existieren, die über die einmalige Erstversorgung (wie die Krisenintervention im Rettungsdienst oder die Notfallseelsorge) hinausgehen. Von 105 antwortenden Personen gaben 37 (35,2 %) an, keine solchen Versorgungs- und Beratungsangebote zu kennen. 82,9 % der befragten Einrichtungen gaben an, dass in solchen Fällen betroffene Familien in ihrer Einrichtung Hilfe suchen. Auch wenn diese Einrichtungen Familien Hilfe anbieten können, so sind es in 82,4 % der Fälle die Gleichen, wie im spezifischen Beispiel des elterlichen Suizids, d. h. in großem Ausmaß die Weitervermittlung. Auch hier stand die Weitervermittlung in heilkundliche Angebote mit 44,1 % an erster Stelle. 7,9 % gaben an, dass in der Region keine spezifischen Einrichtungen zur Weiterverweisung vorhanden sind. Nur 3 % der Befragten gaben an, die Familie an traumaspezifische Angebote verweisen zu können.

Bezüglich der Weitervermittlung gaben 60,3 % der Antwortenden an, dass es für die Familien zu langen Wartezeiten von mehr als 6 Monaten kommt und weitere 6,3 % berichten von langen Wartezeiten nach einem zeitnahen Erstgespräch. Trotzdem hoben 23,5 % der Befragten die gute Vernetzung zwischen den unterschiedlichen Einrichtungen hervor.

## Diskussion

In der durchgeführten Querschnittstudie mit 108 Mitarbeitenden aus Jugendämtern und Beratungsstellen in Bayern wurde deutlich, dass Fälle von Suizid und Suizidversuch Teil der Beratungs- und Betreuungsrealität dieser Mitarbeitenden sind. Auf die Frage, ob die Befragten in ihrer Einrichtung den im Fallbeispiel benannten Menschen Hilfen anbieten könnten, antwortete die große Mehrheit mit „ja“. Die Beratenden begegnen Fällen wie im Beispiel mit hohem Einsatz, was sich dadurch zeigt, dass fast 20 % einen Beratungstermin am selben Tag möglich machen würden, ein weiteres Viertel innerhalb von 2 bis 4 Tagen und immerhin nochmal ein Drittel innerhalb einer Woche. Darüber hinaus machen mehr als drei Viertel der Einrichtungen, die diese Frage beantwortet haben, eine zeitlich und quantitativ unbegrenzte Begleitung möglich. Dies kann als Hinweis darauf gedeutet werden, dass die befragten Fachkräfte neben dem Versorgungsauftrag der Einrichtung den Bedarf für Beratung bei den Betroffenen sehen und diesem auch in der eigenen Einrichtung entsprechen wollen.

Allerdings fällt auf, dass die am häufigsten genannte Art der Hilfe die Weitervermittlung war. Daraus entstehen diverse Problemstellungen: Die Weitervermittlung stellt an sich ein hochschwelliges Angebot da [1, 15, 17], welches den betroffenen Familien in Akutsituationen nicht zugemutet werden sollte. Die darin liegende Problematik wird zusätzlich dadurch erschwert, dass die Zielgruppe der Menschen, die den Suizid(versuch) einer nahen Bezugsperson erlebt haben, einen sowieso schon erschwerten Zugang zum Hilfesystem haben, u. a. durch das mit Suizid assoziierte Stigma [12, 19–21]. Eine weitere Problemstellung bei der Weitervermittlung liegt darin, dass die am häufigsten genannte Kategorie zur Weitervermittlung die der heilkundlichen Angebote (niedergelassene Fachkräfte mit Approbation, klinische Einrichtungen etc.) ist. Daraus resultieren mehrere Herausforderungen, von denen zwei (die langen Wartezeiten und die mangelnde Passgenauigkeit) im Folgenden näher beleuchtet werden sollen. Bezüglich der Wartezeiten bei Weitervermittlung in therapeutische und andere heilkundliche Angebote gaben 66,3 % der Antwortenden an, dass die Familien (nach einem eventuellen zeitnahen Erstgespräch) mit zu langen Wartezeiten von mehr als 6 Monaten konfrontiert sind. Diese Erfahrung bestätigen auch die Ergebnisse von Müller et al. aus 2019 [18]. Die Ausrichtung auf akute Krisenintervention und frühe präventive Verarbeitungsförderung, die nach belastenden Lebenserfahrungen indiziert ist [2, 4], ist somit in diesen Systemen nicht gegeben. Hier scheinen, bedingt durch die langen Wartezeiten, von denen die Fachkräfte berichten, für die frühe Ereignisverarbeitung relevante Zeitfenster möglicherweise ungenutzt oder unbegleitet zu verstreichen. Die heilkundlichen Regelversorgungsstrukturen verantworten ihrem Auftrag und ihrer Kompetenz nach, als auf die Versorgung von psychischen Erkrankungen ausgelegtes System, die Unterstützung beim Vorliegen krankheitswertiger Störungen. Diese liegen allerdings kurz nach Eintritt des traumatischen Ereignisses in aller Regel noch nicht vor. Daneben ist zu hinterfragen, ob aus jedem Schicksalsschlag zwangsläufig eine pathologische Erkrankung erwächst, die einer heilkundlichen Versorgung bedarf. Der Wert einer frühen und spezifischen beraterischen Begleitung und des Faktors Verarbeitungszeit erscheint auch angesichts der aktuellen Empfehlungen [2, 4] bedeutsam. Vor diesem Hintergrund kommt Kompetenzen der Krisenbegleitung im beratenden, nicht psychotherapeutischen/psychiatrischen Feld eine hohe Bedeutung zu. Diese zeigt sich einerseits in der Befähigung, all die Betroffenen zu unterstützen, die trotz hohem subjektivem Leiden kein klinisches Erkrankungsbild entwickeln. Ebenso könnten das Überbrücken von Wartezeiten und die Identifizierung eines heilkundlichen Hilfebedarfs wertvolle Aufgaben im Beratungsfeld sein.

Andere Angebote, zu denen weitervermittelt wird, sind eher unspezifisch oder zumindest für die Kinder unpassend (z. B. Krisendienst Bayern). Dies ist sicherlich auch der Tatsache geschuldet, dass sich in der Fläche wenige spezifische Beratungsangebote befinden. Die bestehenden Angebote befinden sich zumeist in Metropolregionen und richten ihr inhaltliches Angebot maßgeblich auf zentrale Bedürfnisse der suizidgefährdeten Personen selbst aus. Dabei ist die Versorgung der durch die Gewaltfolgen eines Suizids oder Suizidversuchs betroffenen Angehörigen ebenso bedeutsam. Während Erwachsene zumindest noch teilweise ein entsprechendes Angebot erhalten, sind den Befragten in den meisten Regionen keinerlei spezifische Angebote, die sich bspw. direkt an jüngere Kinder und deren Betroffenheit richten, bekannt.

Grundsätzlich wird die externe Angebotslandschaft von den Fachkräften sehr unterschiedlich beschrieben. Obwohl ein Großteil der befragten Fachkräfte in Führungspositionen tätig ist, wodurch eine gute Vernetzung und eine gute Kenntnis der Angebote im Einzugsgebiet zu erwarten sind, waren mehrheitlich keine passgenauen Angebote für die Versorgung von Familien nach traumatischen Ereignissen bekannt. Dies legt nahe, dass das gezeichnete Bild realistisch ist. Positive soll erwähnt werden, dass die gute Vernetzung unter den Akteuren und Einrichtungen von den befragten Fachkräften hervorgehoben wurde. Insgesamt lässt sich aus den zuvor berichteten Ergebnissen kein eindeutiges Bild über die Bewertung der Angebotslandschaft – weder für das Fallbeispiel noch für allgemeinere Fälle – durch die befragten Fachkräfte ablesen.

Interessant ist auch, dass der Anteil derer, die Hilfe anbieten können, für die Kinder geringer ist als für die Mutter und auch zwischen den unterschiedlich alten Kindern nochmals Unterschiede bestehen. Dem jüngeren Kind können weniger Personen Hilfe anbieten als dem älteren. Die Unterschiede können ggf. darauf zurückzuführen sein, dass für die Kinder weniger Hilfen als für Erwachsene zur Verfügung stehen und dass das Alter der Kinder hier auch nochmals eine Rolle spielt (je kleiner, desto weniger Hilfsangebote). Allerdings ist auch bekannt, dass Fachkräfte den Hilfebedarf von kleinen Kindern nach dem gewaltsamen Tod einer Bezugsperson oder anderen traumatischen Erlebnissen deutlich unterschätzen [6, 9]. Die Annahme, dass diese Kinder keine Unterstützung brauchen, entsteht aus der Überzeugungen, dass diese Kinder zu jung sind, um den Tod verstehen und Trauer erleben zu können, dass sie sich an nichts werden erinnern können und dass eventuelle Auswirkungen sich im Laufe ihrer Entwicklung „verwachsen“ werden [9]. Diese Fehlannahmen ignorieren die Tatsache, dass emotionale Komplikationen nach gewaltsamen Toden schon in sehr jungen Kindern eine Realität sind [9] und schon Kleinkinder anfällig für negative Folgen traumatischer Ereignisse sind, da sie nur über begrenzte Bewältigungsfähigkeiten verfügen und in hohem Maße davon abhängig sind, dass ihre primäre Bezugsperson sie körperlich und emotional schützt [6, 23]. Darüber hinaus haben Untersuchungen gezeigt, dass bis zu 50 % der Vorschulkinder, die nach einem Trauma an einer posttraumatischen Belastungsstörung (PTBS) leiden, nicht spontan genesen und die Diagnose mindestens 2 Jahre lang bestehen bleibt [24]. Besondere Relevanz haben diese Ergebnisse vor dem Hintergrund, dass traumatische Erlebnisse in der frühen Kindheit potenziell größere Auswirkungen auf den Entwicklungsverlauf haben, als solche, die im späteren Jugendalter erlebt werden [5].

Unter dem Gesichtspunkt, dass 58,1 % der Fachkräfte angaben, kein spezifisches Wissen zu akutem traumatischen Stress bei Kindern und Jugendlichen erlangt zu haben, lässt sich sowohl schließen, dass es eine Lücke hinsichtlich spezifischer Aus‑, Fort- und Weiterbildung zu akutem traumatischem Stress bei Kindern und Jugendlichen gibt, als auch, dass die Familien (zumindest im ersten Schritt) auf Fachmenschen treffen, die sich trotz allem Wunsch zu helfen, nicht ausreichend für diese Situationen ausgerüstet fühlen. Der Wunsch der Fachkräfte nach vertiefter Aus‑, Fort- und Weiterbildung zum Thema „akuter traumatischer Stress bei Kindern und Jugendlichen“ kann Hinweis auf unterschiedliche Anliegen sein. Zum einen kann das Bedürfnis der Familie selbst besser helfen zu können eine Motivation sein, insbesondere da viele Fachkräfte angeben, keine geeigneten Stellen zu kennen, bei denen die Familie passende, traumaspezifische Hilfe finden könnte. Zum anderen kann auch der Wunsch nach besserer Einschätzung der Belastungssituation der Familie Hintergrund sein, damit eine Weiterleitung zielgerichteter passieren kann. In beiden Fällen wären dies Szenarien, die nicht in den Zuständigkeitsbereich der Mitarbeitenden der Jugendämter fallen und im Rahmen des täglichen Arbeitsalltags weit über personelle und zeitliche Ressourcen hinaus gingen. In Anbetracht der Vielzahl der bereits bestehenden Verantwortlichkeiten und Kompetenzen in den Einrichtungen stellt sich die Frage, wie viele weitere Verantwortlichkeiten und Versorgungsaufträge bei den derzeitigen Ressourcen noch zuzumuten sind. Der geäußerte Wunsch nach einer unmittelbar erreichbaren fachkompetenten Unterstützung sowohl für die Fachkräfte selbst als auch für die betroffenen Familien verdeutlicht zusätzlich die Bedarfe, die aus der Felderfahrung der Jugendämter und Beratungsstellen beschrieben werden.

Abgeleitet aus der Selbsteinschätzung der Fachkräfte zu ihren Kompetenzen im Bereich des akutem traumatischen Stresses bei Kindern und Jugendlichen, dem Wunsch nach mehr Wissen und Kompetenzen und der Einschätzung der Sinnhaftigkeit einer zentralen Notfallrufnummer für Fachkräfte und betroffene Familien, sind verschiedene Möglichkeiten zur Verbesserung der Versorgungssituation denkbar. Neben der Erfassung stellenweise bereits bestehender Versorgungsstrukturen ist auch die Darstellung neuer Möglichkeiten Zielsetzung des Projekts „Kurswechsel“. Im Vorgriff auf den Abschlussbericht seien hier beispielsweise genannt, die zentrale Bereitstellung von: Selbsthilfematerialien und psychoedukativen Inhalten für Betroffene, alterssensitiven Hinweisen zur Belastungseinschätzung bei Kindern und Jugendlichen, spezifischem Beratungsmaterial für Fachkräfte und einer zentralen Notfallrufnummer oder einer zeitnahen und niedrigschwelligen Videosprechstunde.

Die hier vorgestellte Erhebung weist einige Limitationen auf, welche hier offen gelegt werden sollen. Da sich die Telefoninterviewer*innen beim Anruf als solche zu erkennen gaben, erfolgte die Weitervermittlung für die Interviews meist an Mitarbeitende in Leitungspositionen anstatt an die Mitarbeitenden, an die eine anrufende Familie durchgestellt werden würde. Daher überschätzen die Antworten möglicherweise die durchschnittliche Kompetenz und das Strukturwissen der Mitarbeitenden.

Jugendämter und Erziehungsberatungsstellen sind nur zwei der möglichen Anlaufstellen für Familien nach traumatischen Lebensereignissen. Obwohl diese einen gesetzlichen Versorgungs- und Hilfeauftrag für Familien innehaben und somit eine zentrale Rolle einnehmen, gibt es auch andere Anlaufstellen, die in dieser Erhebung nicht mit einbezogen wurden. Eine Erhebung unter anderen Akteur*innen des Hilfesystems könnte ein Thema weiterer Forschung sein.

Einschränkend sollte auch erwähnt werden, dass die telefonische Erhebung zwar nicht während eines COVID-19-Pandemie-bedingten („coronavirus disease 2019“) Lockdowns stattfand, die Erreichbarkeit der Einrichtungen jedoch evtl. trotzdem pandemiebedingt schlechter als sonst gewesen sein könnte. Allerdings erscheint es trotzdem bemerkenswert, dass einige Einrichtungen (darunter auch Jugendämter) während der normalen Geschäftszeiten nicht einmal per Anrufbeantworter erreicht werden konnten.

## Fazit für die Praxis

- Fälle von Suizid und Suizidversuch kommen in der Beratungs- und Betreuungsrealität von Mitarbeitenden in Jugendämtern und Beratungsstellen vor.
- Fachkräfte sehen den Bedarf für Beratung bei den Betroffenen und wollen diesem auch in der eigenen Einrichtung entsprechen, fühlen sich jedoch in Folge begrenzter interner und externer Angebote sowie eigener Qualifikation dafür nicht ausreichend ermächtigt.
- Die am häufigsten genannte Hilfe ist die Weitervermittlung in andere Angebote. Diese Angebote sind in den meisten Fällen weder spezifisch, passgenau noch kurzfristig verfügbar, was angesichts des hohen Erkrankungsrisikos der betroffenen Kinder und Jugendlichen einen weiteren kritischen Faktor darstellt.
- 80 % der befragten Mitarbeitenden in Beratungsstellen und Jugendämtern halten die Einführung einer zentralen Notfallrufnummer für unmittelbar erreichbare fachkompetente Unterstützung sowohl für Familien als auch Fachkräfte für sinnvoll.
